# Exosomes in the tumor microenvironment: Promoting cancer progression

**DOI:** 10.3389/fimmu.2022.1025218

**Published:** 2022-10-06

**Authors:** Ye Jin, Jianming Xing, Kejin Xu, Da Liu, Yue Zhuo

**Affiliations:** ^1^ School of Pharmacy, Changchun University of Chinese Medicine, Changchun, China; ^2^ School of Life Sciences, Jilin University, Changchun, China; ^3^ School of Acupuncture-Moxi Bustion and Tuina, Changchun University of Chinese Medicine, Changchun, China

**Keywords:** exosomes, cancer, tumor microenvironment, metastasis, angiogenesis, immunosuppression

## Abstract

Exosomes, which are extracellular vesicles produced by endosomes, are important performers of intercellular communication functions. For more than three decades, there has been a growing awareness of exosomes as the contents of the tumor microenvironment and their intimate connection to the development of cancer. The composition, generation, and uptake of exosomes as well as their roles in tumor metastasis, angiogenesis, and immunosuppression are discussed in this paper. In order to stop the progression of cancer, it is crucial to find new diagnostic biomarkers and therapeutic targets for the disease. Knowing the biological characteristics of exosomes and their functions in tumor development helps in this endeavor.

## Introduction

In the 1970s, Johnstone discovered vesicle-like lipid particles secreted by cells during the culture of sheep reticulocytes, which were isolated and named as exosomes (1). Exosomes are a type of extracellular vesicles (EV), and based on occurrence, size and biophysical properties, EVs are divided into three main categories, exosomes (30-200 nm), microvesicles (100-1000 nm) and apoptotic vesicles (>1000 nm) (2, 3). In this paper, we mainly discuss exosomes. Initially, exosomes were thought to be waste excreted by cells; however, as research progressed, it was discovered that exosomes are not useless, but rather are important players in intercellular communication. James E. Rothman et al. were awarded the 2013 Nobel Prize in Physiology or Medicine for their work on the mechanisms regulating intercellular vesicular transport. Gradually, exosomes have been found to be associated with many pathological processes (4, 5). Because of this, they are at the forefront of biological research. Exosomes are rich in DNA, RNA, proteins, lipids and other substances, and the composition of exosomes originating from different cells varies considerably (6). Exosomal tRNA-derived small RNAs (tsRNA) are enriched in human esophageal squamous cell carcinoma and have diagnostic and prognostic potential (7). In the study by Shu et al. the proteins of breast cancer cells with epithelial and mesenchymal phenotypes differed, and the two breast cancer subtypes were distinguished by analysis of exosomal protein content (8). Normal alveolar cells are distinguished from lung cancer cells by comparing the Raman spectra produced by lipids and membrane proteins of exosomes (9).Compared to normal tissues, tumor tissues contain more exosomes (6), which is a reflection of the frequent communication between tumor cells. Tumor-derived exosomes (TEXs), with cytokines, chemokines and other extracellular vesicles, form the tumor microenvironment where they assist intercellular communication and further contribute to cancer progression (10).

In this review, we describe the biological properties of exosomes in terms of their origin, uptake processes, and composition, which are the basis for the exercise of intercellular communication by exosomes. We describe the role of exosomes in tumor promotion in terms of metastasis, angiogenesis and immunosuppression. This is enlightening for the development of anti-cancer drugs, diagnosis and prognosis of cancer (11–13). The research on the composition and function of exosomes is still in its early stages, but in the future, the research and application of exosomes will become more in-depth, which may be the key to overcoming cancer.

## Exosome biology

**Figure 1:**
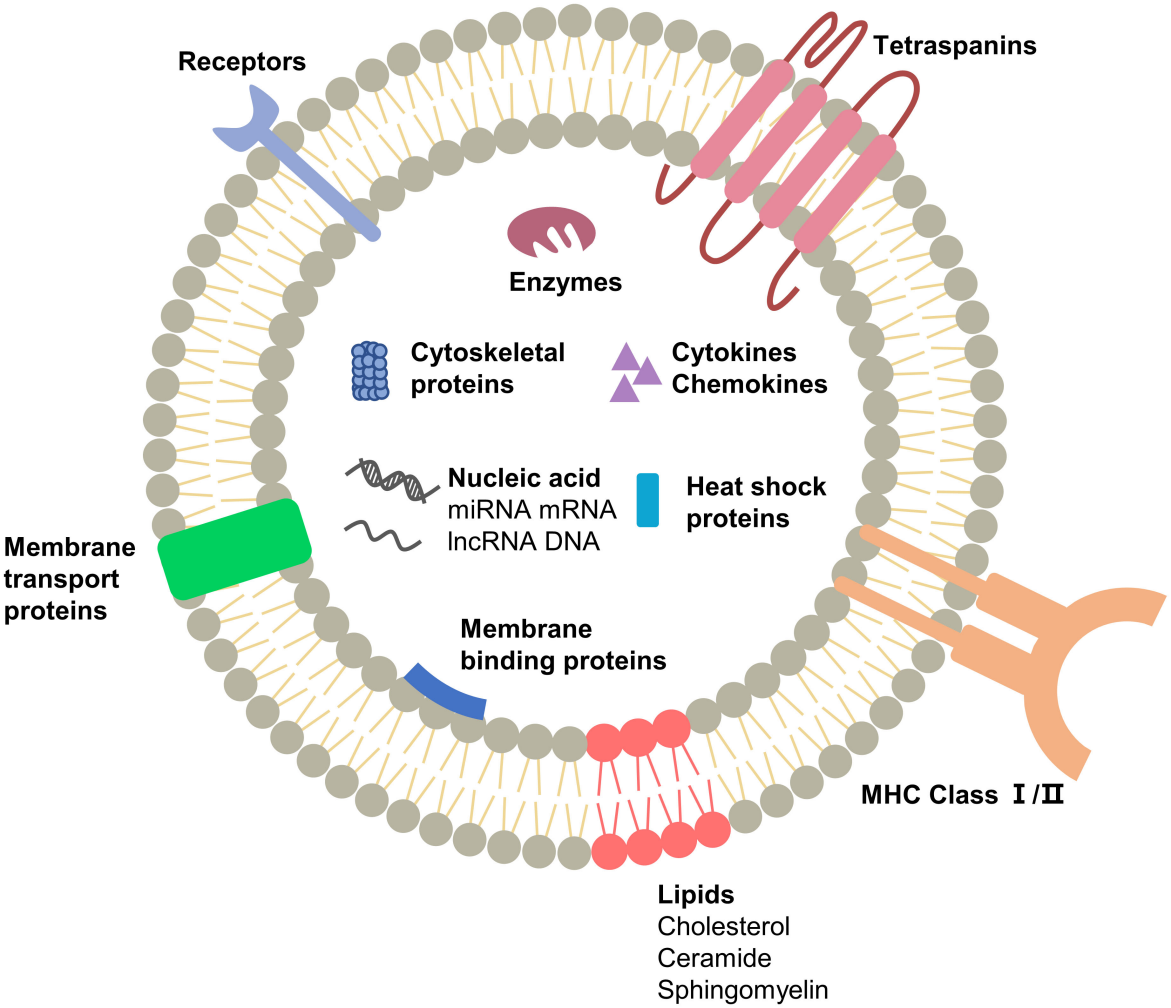
Schematic representation of exosome composition. The main substances are proteins, nucleic acids and lipids, which are the structural and functional basis of exosomes. The components in exosomes secreted by different cells may vary.

Extracellular vesicles are important mediators of proximal and remote communication between cells and mediate intercellular communication through the transmission of genetic information (14). Exosomes are secretory intraluminal vesicles (ILVs) formed by the inward outgrowth of multivesicular vesicles (MVBs) and are one of the three endings of MVBs. The other two types either fuse with lysosomes or recycle through the trans-Golgi network (15). Tumor-derived exosomes (TEXs) contain large amounts of nucleic acids, transcription factors, proteins and lipids (**Figure 1**). TEXs reflect the composition of tumor cells because they are secreted by tumor cells. Compared to exosomes secreted by normal cells, TEXs have more specific proteins and RNAs, which provide a reference for tumor detection (16). Tumor cells can be distinguished from normal cells based on the examination of one of the components of exosomes. MicroRNA is a non-coding RNA approximately 22 nucleotides long, one of the most abundant RNAs in exosomes, which can regulate gene expression by binding to mRNA and inhibiting the translation process (17). MicroRNAs exist stably in body fluids and differ between tumor cells and normal cells, between tumor cells of different origins, and between benign diseases and malignant tumors, so microRNAs are used as biomarkers for cancer diagnosis (18). Taylor et al. found significant differences in exosomal microRNA between patients with benign ovarian disease and those with ovarian cancer, a non-invasive method that could be used for screening for symptoms (19). Other RNA molecules such as mRNAs, ribosomal RNAs (rRNAs), transfer RNAs (tRNAs), long non-coding RNAs (lncRNAs), small nuclear RNAs (snRNAs), and single-stranded DNA and double-stranded DNA molecules have been found in exosomes, and their diagnostic and therapeutic potential for cancer is gradually being discovered (19–23).In addition, proteins play an important role in the functional execution of exosomes. The protein content of TEXs differs from that of exosomes produced by normal cells, and this difference is also associated with different stages of the disease. Exosomes isolated from the plasma of patients with acute myeloid leukemia (AML) have high levels of transforming growth factor-β1 (TGF-β1) and the levels change gradually during chemotherapy (24). Therefore the specific protein content of TEXs provides information for the diagnosis and prognosis of cancer.

**Figure 2:**
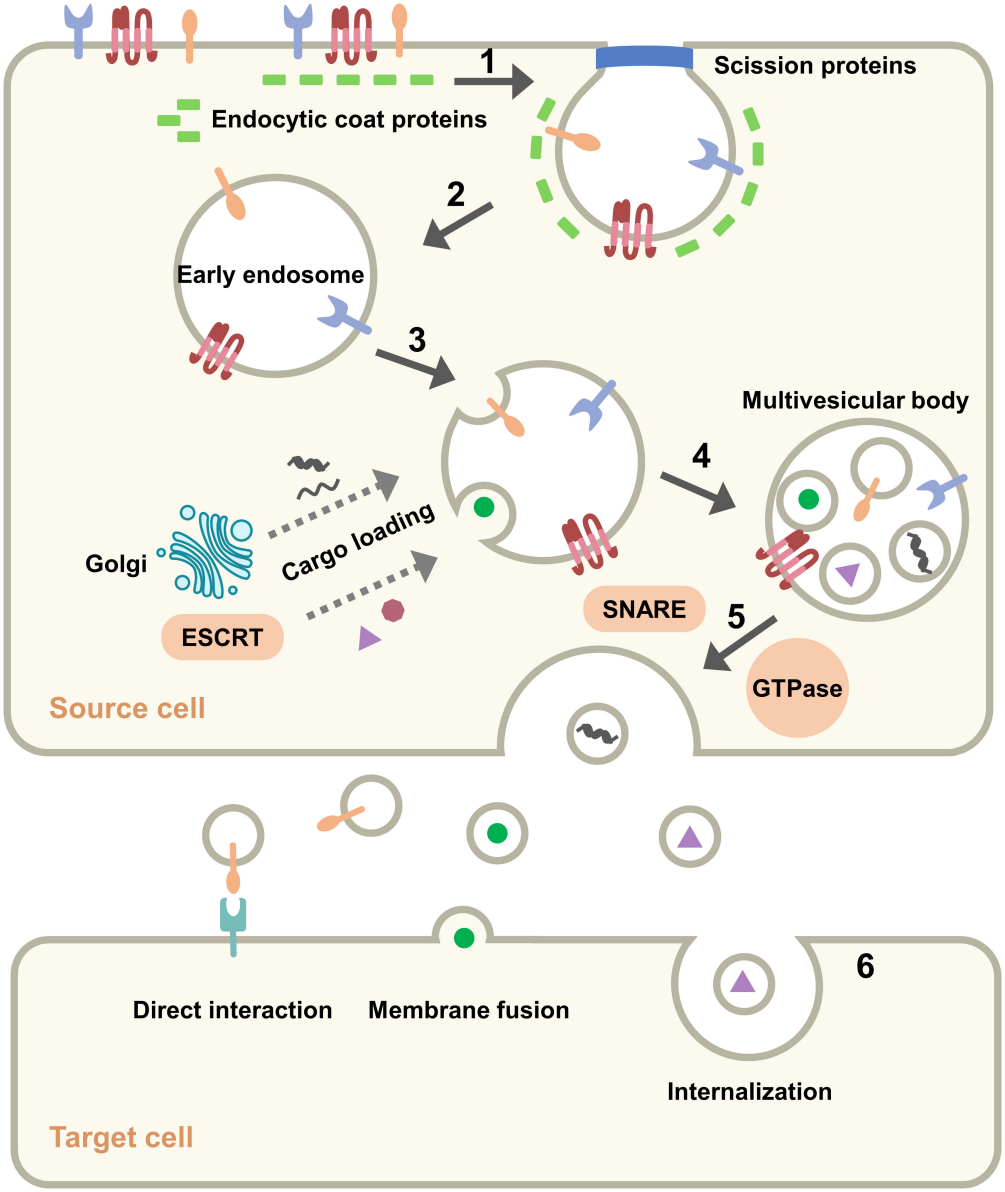
Pathway of exosomes: origin, formation, secretion and uptake. (1) Plasma membrane invagination. (2) Formation of early endosome. (3) Cargo loading and production of intraluminal vesicles. (4) Formation of multiple vesicle bodies. (5) Secretion of exosomes from the source cell to the outside. (6) Mode of exosomes with target cell.

Similar to exosomes secreted by normal cells, some components of TEXs perform duties during exosome movement. Surface components such as tetraspanins, glycoproteins, and adhesions, are significant in the production, transport, and uptake of exosomes. GTPases and ESCRT proteins are involved in component selection and secretion during exosome formation (2). Since exosomes are “messengers” between cells, they contain cytokines, signaling receptors and MHC molecules that can efficiently and accurately activate downstream signals, induce immune responses and enable intercellular communication (20). Another class of components cannot be left out: lipids such as cholesterol, sphingomyelin, phosphatidylcholine, etc. (25, 26), which provide anchoring sites for membrane proteins, encapsulate cargo in a separate space, and enable exosomal activity through fusion and separation. In astrocytes cholesterol was found to regulate exosome release through stimulation of the PI3K/Akt signaling pathway (27). Qi et al. inserted phosphatidylcholine into the exosome to deliver the drug, which effectively improved the uptake rate of the drug (28). It may seem that the components are not related to each other, but the fact is that they work together for the formation, release and uptake of exosomes (**Figure 2**).

Formation of exosomes is a multi-step process. Initially, the plasma membrane remodels and begins endocytosis, which is clathrin-mediated and involves a variety of proteins (29). The endocytic proteins in the cytoplasm accumulate on the inner leaflet of the plasma membrane and recruit other proteins to form a “coating” that bends and invades the plasma membrane. The vesicles are shed by the action of actin and shear proteins and are called early endosomes (30). Endosomes function in a variety of signaling systems through the sorting and transport of ligand-receptor complexes at specific times (31). Subsequently, intraluminal vesicles begin to form in the endosomes, which then become late endosomes or multivesicular bodies (MVBs). Some nucleic acids and proteins are assembled into vesicles *via* the Golgi apparatus or cytoplasm, some cargoes are assembled *via* the endosomal-sorting complex required for transport (ESCRT) machinery. The ESCRT complex consists of about 20 proteins that assemble to form four complexes (ESCRT-0, ESCRT-I, ESCRT-II and ESCRT-III) with associated proteins (VPS4, VTA1, ALIX), and these components collaborate to recognize ubiquitinated proteins, promote vesicle division, and cargo loading (32, 33). Several ESCRT-independent mechanisms based on lysobisphosphatidic acid (LBPA) and ceramides have also been identified, and these lipids help endosomal traps to form vesicles (34, 35). MVBs are transported near the cell membrane and fused to the plasma membrane, driven by Rab GTPase with soluble N-ethylmaleimide-sensitive factor attachment protein receptors (SNARE) complex, and exosomes are secreted outside the cell. Screening of five Rab GTPases that promote exosome secretion, among which Rab27a is associated with the size of exosomes and Rab27b is associated with the regional distribution of multivesicular endosomes (MVEs) (36). Multiple SNARE complexes contribute specificity to the process of membrane fusion, and SNAREs are classified as v-SNAREs and t-SNAREs. The v-SNAREs on MVBs recognize and bind to t-SNAREs on the plasma membrane, allowing membrane fusion and secretion of exosomes (37). When the exosome reaches the target cell, there are three main possible outcomes: (і) activation of downstream signals through ligand binding to receptors on the surface of target cells; (ii) release of cargo by fusion with the plasma membrane of target cells; (ii) release of cargo after internalization by the target cell, dependent on clathrin (38), lipid raft or caveolin (39). The uptake of exosomes differs between cells, and this difference does not depend on the expression of exosome marker proteins but on the recipient cells. Horibe et al. showed that variance in exosomes uptaken by receptor cells was relevant to organ-specific transfer of exosomes (40). Exosomes interact with receptor cells to transmit signals that underlie a series of events such as metastasis, invasion, and immunosuppression in tumor tissue.

## Tumor microenvironment

The tumor microenvironment (TME) is the dynamic multicellular environment in which the tumor exists, and it also contains immune cells, stromal cells, extracellular matrix, growth factors, chemokines, exocysts, blood and lymph (41). It is a vast collection with inhibition and promotion process of tumor development, and it’s perhaps far more complex than imagined. Tumors continue to develop along with the evolution of TME and are influenced by several factors: (i) Cancer cells secrete exosomes, whose active substances produce intercellular effects; (ii) Environmental conditions, such as hypoxia, low pH, and nutritional deficiencies that stress the cells; (iii) Induction of inflammatory and immune reactions (42, 43). Extracellular matrix (ECM), as part of the TME, changes in response to changes in the TME, affecting the downstream signals. The ECM consists of a complex network of macromolecules that provide the basis for the organization of the structure. However, the ECM not only acts as a scaffold for the tissue, but also provides critical biological signals. Cancer cells stiffen the ECM and further produce downstream effects that direct cellular activity and regulate vascular development and immune function (44, 45). ECM further affects the migration of cancer cells by altering their physical properties and composition, and provides an environment for intercellular communication.

Cell-to-cell interaction is an important process in tumor immunotherapy. Inhibitor targeting colony-mulating factor 1 receptor (CSF1R) inhibits transmembrane tyrosine kinase class III receptor, and then inhibits macrophage differentiation and survival, reducing the number of tumor-associated macrophages (TAMs) (41, 46, 47). In addition to designing drugs targeting receptors, cytokine-based drugs have also been the target of ongoing exploration. Interleukin 2 (IL-2), which activates T cells and NK cells, has been used in the clinic for a long time and is approved by the FDA for the treatment of melanoma (48). Researchers have used orthogonal IL-2 cytokines to transmit natural IL-2 This allows for the selective production of desired T cell subsets that hold promise in the cell therapy of cancer (49). In summary, targeting the immune compartment of the TME is the key to tumor therapy, but also the greatest resistance because of the complexity of the tumor microenvironment. In TME, the signaling networks are interconnected and exosomes, cytokines, and chemokines make the whole system constantly and dynamically changing (50).

Cancer cells secrete more exosomes than normal cells, which is inextricably linked to the influence of TME. Conditions such as hypoxia and nutritional deficiency in TME promote the formation and secretion of TEXs (51). TEXs play vital roles in cellular communication between tumor cells and between tumor cells and stromal cells in TME, and these roles are mainly generated through the transfer of cargoes of nucleic acid molecules, lipids, proteins, etc., mainly including: (і) altering normal cells to tumor cells; (ii) inhibiting the immune response of immune cells against tumor cells; (iii) promoting angiogenesis; (iv) stimulating the EMT process and thus promoting metastasis (42). Therefore, studying how TEXs in the tumor microenvironment contribute to tumor development will deepen the understanding of cancer and provide a biological basis for cancer diagnosis and treatment.

## Exosome and metastasis

Metastasis is one of the essential stages of tumor development and a major cause of cancer death. An understanding of metastasis begins with Stephen Paget’s “seed and soil” hypothesis (52): The hypothesis refers to cancer cells that can proliferate malignantly as seeds and the microenvironment suitable for their growth as soil. The metastasis is the result of the action of “seed” and “soil” (52). Metastasis is divided into three main steps: (i) Activation of epithelial-mesenchymal transition (EMT); (ii) Formation of premetastatic niche (53); (iii) Uncontrollable development of cancer cells (54). Exosomes, as part of TME, play an important role in every step of metastasis. The understanding of the role of exosomes in metastasis helps to grasp the process of cancer development and provides more possibilities for cancer diagnosis and treatment (21, 55, 56).

**Figure 3:**
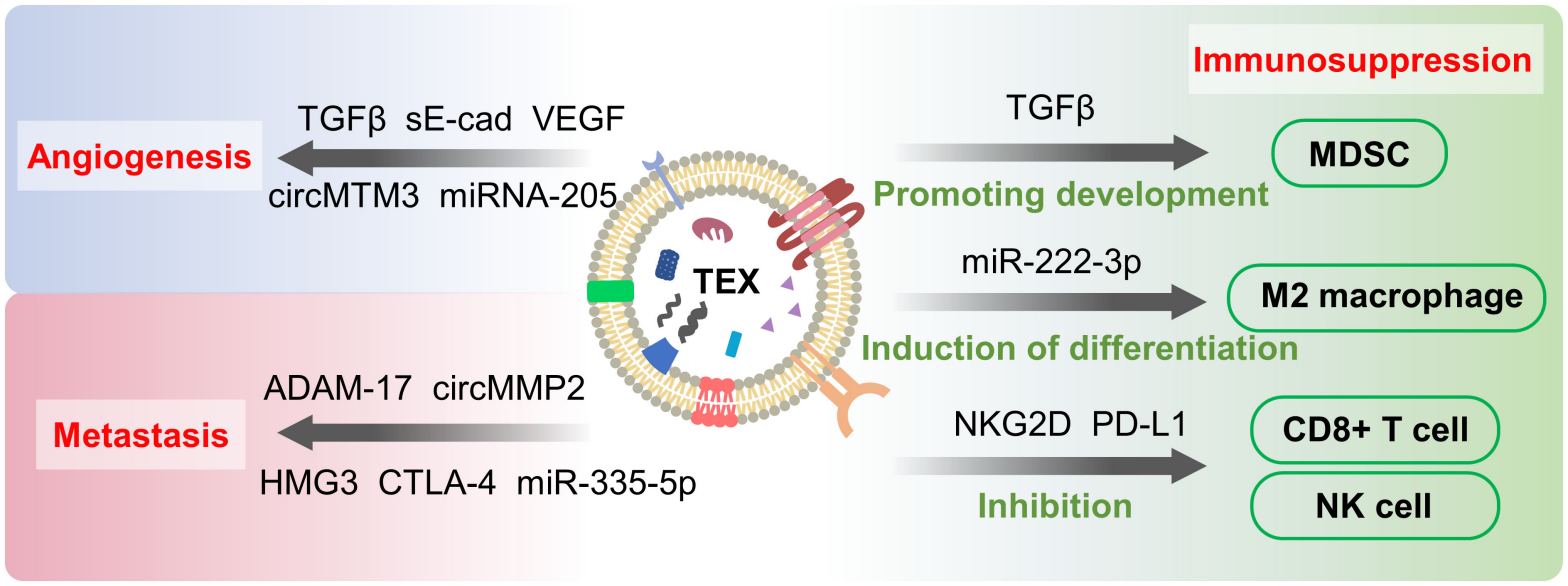
Tumor-secreted exosomes (TEXs)-mediated metastasis, angiogenesis and immunosuppression. TEXs promote angiogenesis, metastasis and immunosuppression by delivering “cargo” such as proteins and nucleic acids to cancer cells and other cellular influences. These processes contribute to the development of cancer and cause changes in TME as well.

The proteins in exosomes are crucial factors affecting metastasis (**Figure 3**). In a study by Xie et al, pancreatic ductal adenocarcinoma (PDAC)-derived exosomes delivered CD44v6/C1QBP complexes to the plasma membrane of hepatic satellite cells and promoted hepatic metastasis of PDAC (57). Nuclear exosomes (nEXOs) are exosomes rich in nuclear proteins and DNA, and are potential tumor markers. nEXOs overexpress high mobility group box 3 (HMGB3), which promotes nasopharyngeal carcinoma metastasis by accelerating angiogenesis, and HMGB3 provides inspiration for finding markers of nasopharyngeal carcinoma metastasis (58). Complex signaling networks in TME influence cancer progression, and exosomal proteins often promote metastasis through signaling pathways. Exosomal cytotoxic T lymphocyte antigen 4 (CTLA-4) promotes hepatocellular carcinoma metastasis by regulating the PTEN/CD44 pathway (59). Similarly, exosomes transport Wnt10b from fibroblasts to breast cancer epithelial cells, which in turn induce EMT to promote cancer progression *via* the Wnt pathway (60). EMT is the initiating step of tumor metastasis, a process in which epithelial cells are transformed into a mesenchymal type and acquire invasiveness. Subsequently, a premetastatic niche is formed through extracellular matrix remodeling as well as angiogenesis (53). Colon cancer cell-derived exosomal ADAM-17, a catabolic integrin and metalloproteinase, promotes colon cancer metastasis by cleaving E-cadherin junctions and participating in the formation of premetastatic niche (61). However, Liu et al. showed that the role of exosomes alone is not sufficient to promote metastasis. Although CD97 is exosome-dependent in the premetastatic niche, there are several soluble components whose functions have not been identified (62). Enzymes are key components of the signaling pathways, and exosomes promote tumor metastasis by delivering enzymes or influencing them. Hepatocellular carcinoma cells secrete exosomes that deliver circ_MMP2 to human hepatocytes and hepatoma cells to increase matrix metalloproteinase 2 levels (63). Exosomes also use lysine oxidase-like 4 (LOXL4) to promote invasion and metastasis of hepatocellular carcinoma cells, and intracellular LOXL4 activates the FAK/Src pathway to promote cancer cell migration (64).

MircroRNA, by binding to mRNA and repressing translation, is often abnormally abundant in cancer, with higher levels of TEX-derived microRNA having an impact on metastasis (65). Metastatic colorectal cancer (CRC)-derived exosomes deliver miR-335-5p to other cancer cells, promoting EMT and thus CRC cell metastasis (66). CRC cell-derived exosomal miR-221/222 plays a role in the formation of premetastatic niche (67). P53 is a tumor suppressor gene and mutated p53 protein is frequently expressed in a variety of cancers (68, 69). In the study by Ju et al., mutations in p53 increased the levels of exosomal miR-21-3p and miR-769-3p, activated fibroblasts, and induced EMT (70). Nonetheless, not all miRNAs are found to be cancer-promoting, and exosomes from urothelial carcinomas (USs) cells can transport miR-128 to inhibit the proliferation of USs cells and promote their apoptosis (71). Other types of RNA in exosomes have also been found to play a role in metastasis (72). Exosomal long non-coding RNAs (lncRNAs) are non-coding RNAs over 200 nucleotides in length, and in pancreatic ductal adenocarcinoma (PADC) cells exosomal lncRNA-Sox2ot binds competitively to miR-200 to promote PADC metastasis (72). The lncRNA FGD5-AS1 involves in the Stat3/NF-κB pathway to stimulate the polarization of M2 macrophages, leading to the proliferation of pancreatic cancer cells (73). Furthermore, exosomal circular RNA has been found to be associated with cancer cell metastasis (22, 74).

## Exosome and angiogenesis

Angiogenesis is an important step in tumor development and metastasis and is usually induced under conditions of hypoxia and nutritional deficiency (75). Angiogenesis is composed of multiple steps: In the beginning, endothelial cells divide rapidly under stimulation and there is an imbalance between activating and inhibiting mechanisms. The vascular basement membrane is degraded, breaking homeostasis and entering the angiogenic phase; Vascular sprouting leading to expansion of the vascular plexus and formation of a network of small and large vessels; Pericytes and smooth muscle cells cover the nascent endothelial channels and become mature vascular network (76, 77). The whole process of angiogenesis consists of regulatory factors acting with stimulatory signals. In addition to the components shed by the extracellular compartment, some of them are delivered to the target *via* exosomes, and exosomes of different origins are all involved in angiogenesis (78).

Hypoxia is one of the characteristics of tumor microenvironment (79), which occurs due to the rapid multiplication of tumor cells and insufficient blood supply. Hypoxia induces the production of large amounts of exosomes, which has been demonstrated in a variety of cancers, but the detailed mechanism is not clear (10, 80). Exosomes carry “cargo” and are internalized by endothelial cells to induce angiogenesis (**Figure 3**). Vascular endothelial growth factor (VEGF) (81), fibroblast growth factor (FGF) (5), and transforming growth factor β (TGF-β) (82) are angiogenesis-stimulating factors frequently carried by exosomes. Soluble E-calmodulin (sE-cad) has also been shown to induce angiogenesis in ovarian cancer, where sE-cad-positive exosomes act on endothelial cells and activate the β-catenin and NFκB signaling pathways (83). Exosomes also affect angiogenesis through the delivery of non-coding RNA. From Li et al, exosomes promote angiogenesis by delivering small nucleolar RNA host gene 16 (SNHG16), a long noncoding RNA that activates the PI3K/Akt/mTOR pathway (84). Exosomal miR-205 was found to accelerate angiogenesis *via* the PTEN-AKT pathway in ovarian cancer (85). Circular RNAs (circRNAs) are single-stranded circular RNA molecules, and a large number of circRNAs have been shown to play a role in cancer (86). For example, the exosomal circular RNA molecule circMTM3 is shown to promote tumor angiogenesis (87). However, recent studies have found that not all non-coding RNAs carried by TEXs are pro-angiogenic. miR-21, although shown to promote angiogenesis through delivery of VEGF, exhibits anti-angiogenic effects in leukemia (88, 89). In addition to TEXs, stem cell-derived exosomes have also been shown to participate in the angiogenic process. Exosomes from MSCs inhibit angiogenesis by downregulating VEGF expression in breast cancer cells (81). An increasing number of studies have shown that exosomes are important players in the angiogenesis of tumor tissues.

## Exosome and immunosuppression

Exosomes produced by tumor cells have been shown to be heavily involved in immunosuppression and promote tumor progression (**Figure 3**). TEX acts on immune cells through programmed death-ligand 1 (PD-L1), transforming growth factor-beta (TGF-beta), fas cell surface death receptor ligand (FasL) and other proteins to cause immunosuppression (6). PD-L1 binds to PD-1 and then suppresses immune suppression by transducing signals to CD8+ cells. Numerous studies have demonstrated that tumor cells upregulate PD1 levels and promote immune escape (90). This process is found to function in breast cancer cells through exosomes carrying PD-1 (91). PD-L1 has also been found on exosomes, and TGF-β directly regulates PD-L1 loading on exosomes in breast cancer, ultimately inhibiting CD8+ cells by controlling the phosphorylation of TCR signaling proteins (92). Exosomes inhibit CD3-ζ and Janus kinase 2 (JAK) expression as well as induce apoptosis of CD8+ T cells through Fas-FasL interaction, reduce CD8+ T cells and increase CD4+ T cells to suppress immunity (93–95). NKG2D is a C-type lectin-like receptor expressed on subpopulations of NK cells, CD8+ T cells and CD4+ T cells that triggers cytotoxicity through the binding of its ligand to target cells. Exosomes expressing NKG2D ligands induce NKG2D downregulation in NK and CD8+ T cells, leading to impaired cytotoxic function (96). Yin et al. revealed that TEXs deliver fatty acids to dendritic cells (DCs), activating the peroxisome proliferator-activated receptor(PPAR) α of DCs in response to excess fatty acids. This reaction shifts metabolism from glycolysis to mitochondrial oxidative phosphorylation, ultimately causing immune impairment in DCs and aiding immune evasion of cancer cells (97, 98). Pan et al. found that exosomal delivery of circNEIL3 to tumor-associated macrophages (TAM) stabilized the oncogenic IGF2BP3 (insulin-like growth factor 2 mRNA binding protein 3) and obtained immunosuppression (99). IGF2BP3 activates PI3K and MAPK pathways to promote glioma cell proliferation, invasion, and chemoresistance (100).

Also of note, TEXs create immunosuppression by promoting the differentiation of immune cells in the direction favorable to tumor. Regulatory T cells (Treg) are suppressor T cells that inhibit the proliferation and activation of CD4+ and CD8+ T cells. TEXs induce Treg production and upregulate the immunosuppressive function of Treg (101). Similarly, TEXs disrupt the differentiation of monocytes to dendritic cells and promote the generation of myeloid immunosuppressive cells (102). It has been demonstrated that TEXs induce the transformation of CD14+ monocytes into myeloid-derived suppressor cells (MDSCs) (103, 104). MDSCs are immature myeloid cells with significant immunosuppressive effects. The main targets of MDSCs are T cells, and MDSCs produce VEGF, basic fibroblast growth factor(bFGF) to influence TME remodeling and angiogenesis, thus promoting tumor progression (51, 105, 106). M2 macrophages are important cells involved in immunity and their function is to suppress inflammation and promote angiogenesis. However, exosomes induce the production of M2 macrophages which promote tumor growth (107) and metastasis (108) as well as stimulate tumor recurrence (109). In epithelial ovarian cancer, exosomal miR-222-3p regulates the SOCS3/STAT3 pathway to induce macrophage differentiation toward the M2 phenotype (110). Exosome-mediated lncRNA HCG18 transfer promotes M2 macrophage polarization in gastric cancer (111).

## Discussion

Cancer is one of the major causes of death and seriously affects human health. However, cancer is not caused by just a group of malignantly proliferating cancer cells but is the result of a combination of multiple stromal cells and signaling molecules. These components coordinate and constrain each other to form the TME and further promote cancer. The exosomes in TME encapsulate proteins, nucleic acids, and other substances to transport cargo from the origin cell to the target cell. The exosomes also matches the surface molecules of the recipient cells to accomplish selective delivery of the signal. Moreover, the high level of exosomes in tumor tissues corroborates more frequent intercellular communication than in normal tissues. This promotes cancer invasion, metastasis, immunosuppression, etc., and provides favorable conditions for cancer development. Although the tumor-derived exosomes are mainly discussed in this paper, however, various stromal cells and immune cells in the tumor microenvironment all secrete different exosomes, forming a complex regulatory network.

The exosomes in the tumor microenvironment reflect the nature of the cells that secrete them, and the molecules carried by TEXs reflect the differences between cancer cells and normal cells. Such differences may be used to find targets for drugs, biomarkers for diagnostics, and tracking of disease progression. The relationship between TME and TEXs is complex, with TME influencing the production and secretion of TEXs, and TEXs influencing tumor processes through cargo delivery, further influencing TME. However, not all components of TEXs are cancer-promoting, and more regulatory processes need to be discovered. Exosomes also play a role in drug resistance, increasing tumor tolerance to drugs and affecting treatment outcomes. In recent studies, various RNAs from exosomes have been found to be involved in tumor drug resistance: lncRNA (112, 113), miRNA (114–116), circRNA (117), etc. They enhance the resistance of tumor cells to chemotherapeutic agents by affecting the production of proteins and by participating in various signaling pathways (118). Treatment using exosomes may be an effective way to fight cancer, such as making drug delivery systems. However, several issues need to be addressed before application: Is there a comprehensive understanding of the composition and function of exosomes? How are immunogenicity and safety determined? How can efficiency and stability be improved during delivery? All of these issues need to be further addressed. Therefore, an understanding of the biological properties and functions of exosomes is essential. This is a prerequisite for effective application and the purpose of this review. Research on the heterogeneity, long-distance delivery, and diverse mediation network of exosomes in TME will provide more references for the clinical application and bring more options for cancer treatment.

## Author contributions

YJ wrote the manuscript and drew the pictures with partial help from JX and KX. DL and YZ edited and revised the manuscript. All authors contributed to the article and approved the submitted version.

## Funding

This work was supported by the National Natural Science Foundation of China (Grant No. 82003985, 81973712), Jilin Province Science and Technology Development Project in China (Grant No. 20210204013YY), Jilin Province Science and Technology Development Plan Project (Grant No. 20200708081YY), and China Postdoctoral Science Foundation (Grant No. 2020M670825, 2020T130568).

## Conflict of interest

The authors declare that the research was conducted in the absence of any commercial or financial relationships that could be construed as a potential conflict of interest.

## Publisher’s note

All claims expressed in this article are solely those of the authors and do not necessarily represent those of their affiliated organizations, or those of the publisher, the editors and the reviewers. Any product that may be evaluated in this article, or claim that may be made by its manufacturer, is not guaranteed or endorsed by the publisher.
